# Digitally excluded: Inequalities in the access and use of online learning technologies in Scottish secondary schools.

**DOI:** 10.23889/ijpds.v7i3.1819

**Published:** 2022-08-25

**Authors:** Morag Treanor, Patricio Troncoso

**Affiliations:** 1 Heriot-Watt University

## Objectives

Our overall aim is to analyse the patterns and impacts of inequalities in the access to and use of digital learning technologies in Scottish secondary schools, especially for young people experiencing socio-economic inequalities.

## Approach

Using a novel dataset arising from the Scholar online learning platform (https://scholar.hw.ac.uk/), which (between June 2020 and June 2021) recorded usage data for c.133,000 students in 339 (95%) publicly-funded Scottish secondary schools, registered for Scottish Qualifications Agency exams: National 5 (age 15/16); Highers (age 16/17) and Advanced Highers (age 17/18). We focused on engagement, measured through average page visits and percentage of active users. We used descriptive statistics and visualisation to understand the patterns of usage across levels and portfolios/subjects, and multilevel negative binomial models to ascertain the extent of the variation across schools and its association with deprivation alongside other characteristics.

## Results

We have found emerging evidence of a socio-economic gradient in the use of digital learning technologies, whereby the students attending the most deprived schools (according to SIMD and FSM-eligibility) show the lowest levels of engagement. Overall, engagement varies substantially across all levels and portfolios/subjects, but it is most noticeably higher in Sciences and in rural areas, which may well be unveiling student needs unmet by rural schools. Focusing on Sciences, variations across schools can be large, highlighting the need to understand what makes students in certain schools engage more or less with online learning. Should the data allow it, we will draw comparisons pre- and post-pandemic to explore the differences in engagement across students and schools.

## Conclusion

In light of a widening socio-economic gap due to COVID-19 restrictions, these findings will help policymakers and practitioners better understand digital inequality, to assess the impacts of policy measures such as providing disadvantaged young people with laptops, to mitigate digital inequality and improve digital services to improve educational outcomes.

